# ﻿Typification of 14 names in the *Dianthusvirgineus* group (Caryophyllaceae)

**DOI:** 10.3897/phytokeys.187.75534

**Published:** 2021-12-13

**Authors:** Gianniantonio Domina, Giovanni Astuti, Gianluigi Bacchetta, Giulio Barone, Ivana Rešetnik, Ana Terlevic, Melanie Thiébaut, Lorenzo Peruzzi

**Affiliations:** 1 Department of Agriculture, Food and Forest Sciences, University of Palermo, Viale delle Scienze ed. 4, I-90128 Palermo, Italy University of Palermo Palermo Italy; 2 Dipartimento di Biologia, Unità di Botanica, Università di Pisa, Via Derna 1, 56126, Pisa, Italy Università di Pisa Pisa Italy; 3 Dipartimento di Scienze della Vita e dell’Ambiente, Centro Conservazione Biodiversità (CCB), Università di Cagliari, V.le S. Ignazio da Laconi 13, 09123, Cagliari, Italy Università di Cagliari Cagliari Italy; 4 Faculty of Science, Department of Biology, University of Zagreb, Marulićev trg 20/II, 10000, Zagreb, Croatia University of Zagreb Zagreb Croatia; 5 Herbier de l’Université Claude Bernard Lyon 1, FR-CERESE, UMR 5023 LEHNA, Lyon, France Université Claude Bernard Lyon France

**Keywords:** *Dianthus*, France, Italy, Slovenia, nomenclature

## Abstract

The nomenclature of 14 taxa from Central and Southern Europe within the *Dianthusvirgineus* group is discussed. *Dianthusaggericola* Jord., *D.collivagus* Jord., *D.consimilis* Jord., *D.orophilus* Jord., *D.saxicola* Jord., *D.juratensis* Jord. are here lectotypified by specimens from the Jordan herbarium in LY, while *D.godronianus* Jord. by a specimen in P. *Dianthussubacaulis* Vill. is neotypified by a specimen collected on Mont Ventoux (S. France) and housed in MPU. For *D.sylvestris* Wulfen, a lectotype is here designated and its previous neotypification is discussed. Dianthuscaryophyllusvar.tenuifolius Moris, D.caryophyllusf.minor Moris and D.sylvestrisvar.garganicus Ten. are lectotypified by specimens housed in herbarium Moris (TO) and herbarium Tenore (K). Dianthusvirgineusvar.tergestinus Rchb. is lectotypified by a drawing from the *Icones florae Germanicae* & *Helveticae*, while D.contractusvar.evolutus Lojac. is neotypified by a specimen in P. For each taxon the currently accepted name is provided including new synonymies. The type indication is followed by nomenclatural and taxonomic notes, in which the original material found is commented and the reasons for the identification of the types are discussed.

## ﻿Introduction

*Dianthus* L. (Caryophyllaceae) includes about 300 species from the temperate regions of the Old World, many of which are narrow endemics (Hardion et al. 2020). This genus still shows challenging systematics. A large part of recent taxonomic research, in fact, has been focused on the description of new taxa. Since 2000, 64 new species and subspecies have been described for the Euro-Mediterranean area, and a total of 98 new names have been published (IPNI 2021). Conversely, the taxonomic contributions on *Dianthus* that have taken into consideration groups of species with statistical analyses of morphological data or molecular investigations are very few (Domina et al. 2017; Hardion et al. 2020). Furthermore, the genus-level taxonomic treatments date back to more than 50 years ago (Williams 1890, 1893; Pax and Hoffmann 1934; Reeve 1967), and the recently published treatments of *Dianthus* in national flora (Bernal et al. 1990; Tison and de Foucault 2014; Vangjeli 2015; Brullo and Guarino 2017; Nikolić 2020) have not brought any significant change to the taxonomy of this genus. In several cases, the nomenclatural types for these taxa are not yet designated (Domina et al. 2021). This created a situation of taxonomic uncertainty. *Dianthus* is an interesting genus, both from a biological and economical point of view (Hardion et al. 2020). Hence, an integrated approach to the taxonomy of this genus is needed.

The *D.virgineus* L. group can be considered as one of the richest and most complex in the genus. Many taxa have been described from Central and Southern Europe, albeit their actual taxonomical value is often doubtful. The genus has undergone extensive taxonomic investigations since the 18^th^ century (Smith 1794), but in many cases the original material used for the description of the taxa is not known and the nomenclatural types have not been designated yet. The lectotype of *Dianthusvirgineus* L., the oldest available name that applies to wild plants in this group, has been designated only recently (Domina et al. 2021). The nomenclature and morphology of the large part of taxa described from Southern Italy, Sardinia, and Sicily have been investigated by Bacchetta et al. (2010). Other typifications were published by Camarda and Corrias (1987), Brullo et al. (2000), Arrigoni (2006), and Clementi et al. (2015). However, most of the taxa described in this group are still to be investigated.

In this study, the nomenclature of 14 taxa is discussed: *Dianthusaggericola* Jord., *D.collivagus* Jord., *D.consimilis* Jord, *D.godronianus* Jord., *D.orophilus* Jord., *D.saxicola* Jord., *D.juratensis* Jord., and *D.subacaulis* Vill. from S. France; *D.sylvestris* Wulfen from NE Italy/SW Slovenia; D.virgineusvar.tergestinus Rchb. from NE Italy; D.caryophyllusvar.tenuifolius Moris and D.caryophyllusf.minor Moris from Sardinia; D.sylvestrisvar.garganicus Ten. from S Italy; and D.contractusvar.evolutus Lojac. from Sicily.

As part of an ongoing project aimed to push forward the taxonomic knowledge on selected genera of the Italian vascular flora, this study aims to lay the foundations for further taxonomic investigations by an integrated approach based on morphometric and molecular data (Domina et al. 2021; Giacò et al. 2021).

## ﻿Material and methods

We examined the scientific literature for the effective place of publication of surveyed *Dianthus* names described from Central and South Europe. The bibliographic data was searched in the available digital sources and in the libraries of the European institutions, while the original material of the investigated species was searched in the main European herbaria: B, C, FI, G, K, MA, MPU, NAP, P, PAL, RAB, RO, TO, W, WU, and ZA; herbarium acronyms follow Thiers (2021). A start-up online screening was possible thanks to digital herbarium specimens’ images provided by GBIF (https://www.gbif.org), Jstor (http://plants.jstor.org), and ReColNat (https://www.recolnat.org/fr/). More thorough investigations were conducted in the Jordan herbarium at LY. The articles of the International Code of Nomenclature for Algae, Fungi, and Plants (hereafter ICN) follow Turland et al. (2019). Until more in-depth, integrated morphometric, genetic, karyological, and ecological information comes to light, our judgments should be considered provisionally accepted, according to current knowledge. In this group, the characters that have been proven to best discriminate species (Bacchetta et al. 2010) are: leaf length and width, number of flowers per scape, shape and length of outer and inner bracts. These characters have been used to check the morphological features of the selected types. The anther and petal length have been proposed as additional discriminant characters, but these can be easily appreciated on fresh plants and not on herbarium samples.

## ﻿Typification of the names

### ﻿ Dianthus aggericola

Taxon classificationPlantaeCaryophyllalesCaryophyllaceae

Jord. in Billot, Annot.: 48. 1856. [December 1856]

6E5DC971-4655-59E9-8816-8397A9FAA569

= D.virgineus L., Sp. Pl. 1: 412. 1753. Ind. Loc.: “du Reculet (Ain)”.

#### Type.

(lectotype here designated): *Dianthusaggericola* Jord., du Reculet, 8 July 1854 [A. Jordan], LY0079734!

#### Note.

No other original material was found in the surveyed herbaria. According to the label, this plant was originally collected in Reculet and then grown in Jordan’s garden, where it was collected in July. As a matter of fact, besides his huge herbarium and library, Alexis Jordan owned a one-hectare experimental garden. We know that he used it to sow most species every year, while maintaining alive perennial ones, and that he regularly made herbarium sheets from these cultivated plants. In this context, the notion of original material requires particular attention (Thiebaut and Tison 2016).

The lectotype designated here matches the protologue and corresponds to the current application of the name, which is considered a heterotypic synonym of *D.godronianus* Jord. in Kerguélen (1993), in turn, currently considered a heterotypic synonym of *D.virgineus* (Domina et al. 2021). The lectotype of *D.aggericola* and that of *D.virgineus*, have the same leaf length and width, uniflowered scapes, the same length and shape of outer and inner bracts, the same calyx length and shape. We, therefore, confirm this synonymy.

### ﻿ Dianthus caryophyllus var. tenuifolius

Taxon classificationPlantaeCaryophyllalesCaryophyllaceae

Moris, Fl. Sardoa 1: 231. 1837. [April 1837]

7BE636BE-F1EC-544D-AE62-827F7771D9A9

≡ D.siculussubsp.tenuifolius (Moris) Arrigoni, Parlatorea 7: 20. 2005.
= D.genargenteus Bacch., Brullo, Casti & Giusso, Nordic J. Bot. 28(2): 145. 2010. Ind. Loc.: “In sterilibus frequens [Sardinia]”.

#### Type.

(lectotype here designated): *Dianthuscaryophyllustenuifolia*, prope Belvì, July, inter rupe / Mus. Bot. Horti Taurinensis, Herb. Moris Barbey Cat Sard. N.156, TO!

#### Note.

Three herbarium sheets are kept in TO, with several individuals each. All three specimens bear labels handwritten by Moris but lack the year of collection. Two of them come from generic localities (“in arenis maritimis” and “in collibus”), while one is from Belvì in the centre of Sardinia (Nuoro). All the specimens are complete and in good condition but refer to different collections: two specimens have been collected in inner localities, whereas another one comes from the coast. Moris reports that the scape bears a single flower and that another taxon (D.caryophyllusvar.tenuifoliusf.minor) grows in arenosis maritimis [sandy coast]. Thus, here we propose the specimen from Belvì as lectotype, despite not being dated, assuming that the herbarium in TO hosts the original material by Moris as already done by Arrigoni (1979), Rizzotto (1989), Escobar García et al. (2010) in other similar cases,

Based on the specimen in TO coming from the coast (referring actually to f.minor), Valsecchi (1985), and then Bacchetta et al. (2010), refer D.caryophyllusvar.tenuifolius to *D.morisianus* Vals. Based on the diagnosis and the lectotype designated here, D.caryophyllusvar.tenuifolius does not belong to *D.morisianus*. The former taxon shows short scapes bearing one or few flowers and epicalyx scales with mucro 0.5–1.5 mm long, while the latter shows longer multiflowered scapes with epicalyx scales with mucro 2.0–3.5 mm long. This interpretation agrees with Arrigoni (2010). According to the lectotype of D.caryophyllusvar.tenuifolius, which shows woody stocks contracted with branches, epicalyx scales with an evident mucro, and small calyx, this taxon is a heterotypic synonym of *D.genargenteus* Bacch., Brullo, Casti & Giusso.

### ﻿ Dianthus caryophyllus f. minor

Taxon classificationPlantaeCaryophyllalesCaryophyllaceae

Moris, Fl. Sardoa 1: 231. 1837. [April 1837]

1499B88A-5717-5935-A771-1F57EA1A53B7

= D.morisianus Vals., Boll. Soc. Sarda Sci. Nat. 24: 333. 1985. Ind. Loc.: “In arenosis maritimis [Sardinia]”.

#### Type.

(lectotype here designated): Dianthuscaryophyllusvartenuifolia, in arenosis maritimis, S. Nicolai flumini major Majo junio / Mus. Bot. Horti Taurinensis, Herb. Moris Barbey Cat Sard. N.156, TO!

#### Note.

A single sheet was found in TO. Albeit it may represent the holotype, it is cautiously designated here as a lectotype.

The selected specimen, uniflorous, has fixed seven portions of plants whose leaves and flower scapes are smaller than those of the typical form. All other characters of the flowers correctly match the protologue. This taxon is a heterotypic synonym of *D.morisianus*, a species described by Valsecchi (1985) for the same area and habitat (Peruzzi et al. 2015), that shows the same leaf length and width and, albeit with multiflowered scapes, the same length and shape of outer and inner bracts, and the same calyx length and shape.

### ﻿ Dianthus collivagus

Taxon classificationPlantaeCaryophyllalesCaryophyllaceae

Jord. in Billot, Annot.: 46. 1856. [December 1856]

6D682F70-C6DB-50D5-B87C-9F203D4F9AB2

≡ D.caryophyllusvar.collivagus (Jord.) Cariot & St-Lager Étude Fl., éd. 8, 2: 104. 1889.
= D.sylvestris Wulfen in Jacq., Coll. 1: 237. 1786. [January-September 1786]. Ind. Loc.: “abonde sur les côteaux du Rhône près de Lyon”.

#### Type.

(lectotype here designated): *Dianthusscheuchzeri* Rchb., *Dianthussylvestris* auct. Gall. ex parte non Wulf, Lyon a Néron, Jordan, odor levis, folia ramis trigemina semper angustissima; *Dianthusscheuchzeri* Jord. non Rchb., *Dianthuscollivagus* Jord., Lyon à Neron, ex herbis Jordan, July 1854, CLF056818!

#### Note.

Other six specimens collected by Jordan are preserved at LY, but they are not original material, since they are lacking a date or reporting dates later than the protologue.

The lectotype designated here matches the protologue and corresponds to the current application of the name, which is considered as a heterotypic synonym of D.sylvestrissubsp.sylvestris in Kerguélen (1993). The lectotype of *D.collivagus*, concerning the shape of calyx teeth, is very similar to the lectotype of *D.inodorus* (L.) Gaertn., which in turn is currently included within the variability of *D.sylvestris* (Domina et al. 2021).

### ﻿ Dianthus consimilis

Taxon classificationPlantaeCaryophyllalesCaryophyllaceae

Jord. in Billot, Annot. 47. 1856. [December 1856]

12424DF9-66CF-5F95-AB07-5AB019AE8CDA

≡ D.caryophyllusvar.consimilis (Jord.) Rouy & Foucaud in Rouy, Fl. Fr. 3: 195. 1896.
= D.sylvestris Wulfen in Jacq., Coll. 1: 237. 1786. [January-September 1786]. Ind. Loc.: “Alpes de l’Oisans”.

#### Type.

(lectotype here designated): *Dianthusconsimilis* Jord., June-July 1855, [A. Jordan] Roux, Herbier Jordan, LY0079676!

#### Note.

At LY we found another specimen citing “Lautaret (H. Alpes, May, June-July 1855, ex Horto Alexis Jordan, LY0079674!” but lacking basal leaves.

The lectotype designated here matches the protologue and corresponds to the current application of the name, which is considered as a heterotypic synonym of D.sylvestrissubsp.sylvestris in Kerguélen (1993). The lectotype of *D.consimilis*, concerning the shape of calyx teeth, is very similar to the lectotype of *D.inodorus* (L.) Gaertn., which, in turn, is currently included within the variability of *D.sylvestris* (Domina et al. 2021).

### ﻿ Dianthus contractus var. evolutus

Taxon classificationPlantaeCaryophyllalesCaryophyllaceae

Lojac., Fl. Sicul. 1(1): 165. 1888. [September 1888]

6EF94FA0-F71A-5444-8177-D57B78D1720E

= D.arrostoi C.Presl, Delic. Prag. 60. 1822. Ind. Loc.: “Sulle più alte vette delle Nebrodi sui terreni ghiaiosi o sulle rupi calcaree di Serre di Quacedda. Juntera Minà Pal!”.

#### Type.

(neotype here designated): *Dianthuscontractus* Jan., *Dianthusconstrictus* Janka, In asperis calcareis elatioribus montis Nebrodes, Julio, M. Lojacono Pojero, P05052873 (photo!).

#### Note.

Neither the original material nor traces of this taxon were found in the herbaria consulted and among the documents accompanying the centuries distributed by Lojacono (Aghababyan et al. 2012; Domina et al. 2014). We chose to designate as a neotype the single specimen found, which is at least collected by Lojacono.

The neotype designated here matches the protologue and allows to consider this name as an heterotypic synonym of *D.arrostoi* C.Presl. Compared to the lectotype of *D.contractus* designated by Bacchetta et al. (2010: 151: s.l., s.d., Jan, NAP-GUSS!), and to the lectotype of *D.arrostoi* designated by Camarda and Corrias (1987: 417), this variety differs only by the more elongated scapes.

### ﻿ Dianthus godronianus

Taxon classificationPlantaeCaryophyllalesCaryophyllaceae

Jordan in Mém. Acad. Roy. Sci. Lyon, Sect. Sci., ser. 2, 1: 241. 1851. [January 19851]

C39F6939-EE2B-573B-9B27-8F77ACEABF96

≡ D.caryophyllussubsp.godronianus (Jord.) P.Martin, Soc. Ech. Pl. Vasc. Eur. Bassin Médit. 19: 93. 1984.
≡ D.sylvestrisvar.godronianus (Jord.) Kerguélen, Lejeunia, Nouv. Sér., 120: 81. 1987.
= D.virgineus L., Sp. Pl. 1: 412. 1753. Ind. Loc.: Coteaux stériles de la région des oliviers. Provence, Hyères, Marseille, Toulon, Apt, mont Ventoux, Vaucluse, Villeneuve; Dauphinée, Rabou près de Gap, Valence, Avignon, Languedoc, Viviers, pont du Gard, Uzés, Montpellier, Mende, Perpignan Corse, Calvi, Bastia, Cervione, Evisa, Otta, Campitello.

#### Type.

(lectotype here designated): Soleirol, Herb. Cors., 959 *Dianthusvirgineus* L. (Gren. et Godr.), *Dianthussylvestris* Duby, Bastia - mai 1823, P05000349 (photo!).

#### Note.

– Jordan (1851, 1856) believed that the plants referred by Godron (1847, 1848) to *D.virgineus* L. actually represent a different species, which he renamed *D.godronianus*. According to Godron (1848), this species grows in the surroundings of Montpellier, South France, and Corsica. A duplicate of the collection no. 959 by Soleirol, explicitly cited as seen by Godron (1848), was chosen as lectotype.

This specimen corresponds with the protologue and with the current application of the name. In Kerguélen (1993), this taxon is considered accepted at varietal rank (D.sylvestrissubsp.longicaulisvar.godronianus). In Jauzein (2014), this taxon is instead included in D.caryophyllussubsp.longicaulis (Ten.) Arcang., but the author argues that it could constitute a distinct subspecies (D.caryophyllussubsp.godronianus (Jord.) P.Martin). *Dianthusgodronianus* is instead considered a distinct species by Tison and de Foucault (2014), although these authors note that some coastal populations in Provence differ for a few morphological features. According to the lectotype features and the recent lectotypification of the latter name (Domina et al. 2021), this species can be regarded as a heterotypic synonym of *D.virgineus*.

### ﻿ Dianthus orophilus

Taxon classificationPlantaeCaryophyllalesCaryophyllaceae

Jord. in Billot, Annot.: 43. 1856 [December 1856]

BE333278-87FC-553B-AF6C-BA6C8E679189

≡ D.caryophyllusvar.orophilus (Jord.) Rouy & Foucaud, Fl. Fr. 3: 195. 1896 [July-August 1896]
= D.sylvestris Wulfen in Jacq., Coll. 1: 237. 1786. [January-September 1786]. Ind. Loc.: “schistes au Lautaret et dans le province de Maurienne (Savoie)”.

#### Type.

(lectotype here designated): *Dianthusorophilus*, Dianthussylvestrisanvar.gracilior, du Lautaret May [18]53-June [18]55 […], LY0825955!

#### Note.

Two syntypes from Col de Lautaret are housed at LY: LY0825955 and LY0087623, both in good condition. We have designated here the most complete one as lectotype. The selected type comes from Jordan’s garden, where it was cultivated since its first collection in 1853.

This specimen conforms to the description of the protologue and corresponds to the current application of the name, which is considered as a heterotypic synonym of D.sylvestrissubsp.sylvestris in Kerguélen (1993). The lectotype of *D.orophilus* concerning the shape of calyx teeth, is very similar to the lectotype of *D.inodorus* (L.) Gaertn., which in turn is currently included within the variability of *D.sylvestris* (Domina et al. 2021).

### ﻿ Dianthus saxicola

Taxon classificationPlantaeCaryophyllalesCaryophyllaceae

Jord., Pugill. Pl. Nov.: 29. 1852 [October 1852]

07A3FFBD-8CF9-587A-A721-A043FFE4FD45

≡ D.caryophyllusvar.saxicola (Jord.) Cariot & St-Lager, Étude Fl., éd. 8, 2: 103. 1889.
= D.sylvestris Wulfen in Jacq., Coll. 1: 237. 1786. [January-September 1786]. Ind. Loc.: “in lapidosis et rupestribus calcareis Beugesi et Delphinatus prope Lyon ubi eum legi”.

#### Type.

(lectotype here designated): *Dianthussaxicola* Jord., Serrières (Ain) près de Lyon, 7 June 1852, A. Jordan, LY0682162!

#### Note.

Two specimens belonging to the original material are housed at LY: LY0682162 and LY0088790. Both are in good condition. We have designated here the most complete one as the lectotype.

This specimen conforms to the description of the protologue and corresponds to the current application of the name, which is considered a distinct species by Tison and de Foucault (2014). The lectotype of *D.saxicola* has 10–15 cm long basal leaves and multiflorous scapes; concerning the shape of calyx teeth, it is very similar to the lectotype of *D.inodorus* (L.) Gaertn., which in turn is currently included within the variability of *D.sylvestris* (Domina et al. 2021). Further research is needed to clarify the relationships between these two taxa.

### ﻿ Dianthus juratensis

Taxon classificationPlantaeCaryophyllalesCaryophyllaceae

Jord. in Billot, Annot.: 47. 1856. [December 1856]

D8A052A9-1CFD-58CC-B2FF-17C609CB2326

≡ D.caryophyllusvar.juratensis (Jord.) Gren., Fl. Chaîne Jurass.: 105. 1865.
= D.sylvestris Wulfen in Jacq., Coll. 1: 237. 1786. [January-September 1786]. Ind. Loc.: “du Mont Reculet (Ain)”.

#### Type.

(lectotype here designated): *Dianthusjuratensis* Jord., mont Reculet (Ain), 24 August 1854, [A. Jordan], LY0083755!

#### Note.

Another herbarium sheet (LY08259243) is preserved at LY; it contains plants collected in 1855 in Villeurbanne, where they were cultivated after being originally collected in the wild at Reculet (Ain).

The lectotype designated here matches the protologue and corresponds to the current application of the name, which is considered as a heterotypic synonym of D.sylvestrissubsp.sylvestris in Kerguélen (1993). The lectotype of *Dianthusjuratensis*, concerning the shape of calyx teeth, is very similar to the lectotype of *D.inodorus* (L.) Gaertn., which in turn is currently included within the variability of *D.sylvestris* (Domina et al. 2021).

### ﻿ Dianthus subacaulis

Taxon classificationPlantaeCaryophyllalesCaryophyllaceae

Vill., Hist. Pl. Dauphiné 3(2): 597. 1789. [September-October 1789]

57BCE7E3-6B23-59E4-9292-2DA96F71CB15

≡ D.sylvestrisvar.subacaulis (Vill.) W.D.J.Koch, Syn. Fl. Germ. Helv. 1: 97. 1835.
≡ D.virgineusvar.subacaulis (Vill.) Ser., Prodr. [A. P. de Candolle] 1: 361. 1824.
≡ D.pungenssubsp.subacaulis (Vill.) Bernal, Laínz, Muñoz Garmendia & Pedrol, Anales Jard. Bot. Madrid 44(2): 571. 1987.
= D.sylvestris Wulfen in Jacq., Coll. 1: 237. 1786. [January-September 1786]. Ind. Loc.: “aux environs du Buis, sur le Mont Ventoux”.

#### Type.

(neotype here designated): Herbier A. Dubuis, DianthussubacaulisVill.subsp.subacaulis, Pentes rocailleuses dénudées près du somment du Mont Ventoux (1912 m). (Vaucluse), 7 July 1955, MPU329773 (photo!).

#### Note.

No original material was found in GRM and in the other surveyed herbaria. Also A. P. V. Mutel’s Herbarium was checked because he used to include Villars specimens in his own herbarium (M. Lefebvre, pers. comm.).

The neotype designated here matches the protologue and corresponds to the current application of the name, which is accepted by both Kerguélen (1993) and Tison and de Foucault (2014). This species is characterized by having 1 cm long basal leaves, very short, 1–5 cm long single-flowered scapes and epicalyx scales lanceolate with a linear mucro. Concerning the shape of calyx teeth, it is very similar to the lectotype of *D.inodorus* (L.) Gaertn., which in turn is currently included within the variability of *D.sylvestris* (Domina et al. 2021). Further research is needed to clarify the relationships between these two taxa.

### ﻿ Dianthus sylvestris

Taxon classificationPlantaeCaryophyllalesCaryophyllaceae

Wulfen in Jacq., Coll. 1: 237. 1786. [January-September 1786]

CAE1C149-79A3-5BA7-AE6B-F258BCD3A9D1

≡ D.caryophyllussubsp.sylvestris (Wulfen) Rouy & Foucaud, Fl. France 3: 193. 1896. Ind. Loc.: – “in montibus illis prope Ponewitsch Baronis Wolkensberg in Carniolia, tum in M. Utocsek prope Pillichgraz; in iis. Vallis Rablensis; denique & in iis Vallis Canalensis &c.”.

#### Types.

(lectotype here designated): The water-coloured iconography published by Jacquin (1781–1786, t. 82, the small individual on the right).

#### Note.

The iconography designated by Bacchetta et al. (2010) as neotype is actually part of the original material as uncited illustration (Art. 9.12 of the ICN), since Jacquin’s Icones and Collectanea work are interrelated. Therefore this neotypification must be corrected in lectotypification. This illustration depicts two individuals: one small with a 2 branched single-flowered stem and one large, unbranched but with multiflowered stems and basal leaves three times longer, exemplifying morphological variation in this species. In the protologue, it is clearly stated that the larger plant was seen only once in Monte Re, near Lake of Predil, NE Italy (“Uno duntaxat, quod miratus sum, loco Montis regii Rablensis, giganteum inveni, caulibus cubitalibus bi- & trifloris”), while smaller plants are common elsewhere in Carniola. Accordingly, we can conclude that the two drawings depict plants originating from two different areas, thus belonging to two different gatherings. Consequently, the type designated by Bacchetta et al. (2010: 143), neotype or lectotype, belongs to more than one gathering and cannot be accepted as a type (Art. 8.1, 8.2, 9.3 of the ICN). Thus, the name remains to be typified. No other original material for this name exists (de Langen et al. 1984), so that we select here as lectotype only the small specimen of the water-coloured iconography published by Jacquin at table 86 that better fits the description “folia ... pollicari aut circiter longitudine… Caulis subquinquepollicaris... Flos plerumque unicus [Leaves … one inch or about one inch long, stem less than 5 inches ... flower generally single]”.

The lectotype here selected agrees with the current application of the name by numerous authors, e.g., Kerguélen (1993), Bacchetta et al. (2010), Tison and de Foucault (2014), Brullo and Guarino (2017), who consider *D.sylvestris* as an accepted species. The overall size of the plant, and the length of the leaves are not stable characters for taxonomic discrimination. The shape and relative size of calyx and epicalyx scales are better discriminating taxonomic characters and are evident in the lectotype. These features allow to distinguish D.sylvestrissubsp.sylvestris from D.sylvestrissubsp.tergestinus (Bacchetta et al. 2010).

### ﻿ Dianthus sylvestris var. garganicus

Taxon classificationPlantaeCaryophyllalesCaryophyllaceae

Ten., Fl. Napol. Syll.: 208. 1831. [July-August 1831]

51CA0E78-BDFB-58F0-94E9-42CFAE26AF79

≡ D.caryophyllussubsp.garganicus (Ten.) Grande, Boll. Soc. Bot. Ital. 1912: 178. 1912.
≡ D.caryophyllusvar.garganicus (Ten.) Fiori, Nuova Fl. Italia 1: 512. 1924.
≡ D.sylvestrissubsp.garganicus (Ten.) Pignatti, Giorn. Bot. Ital. 107: 211. 1973.
≡ D.garganicus (Ten.) Brullo, Braun-Blanquetia 2: 31. 1988.
= D.tarentinus Lacaita, Nuovo Giorn. Bot. Ital. n.s., 18(4): 511. 1911. Ind. Loc.: “Gargano”.

#### Type.

(lectotype here designated): *Dianthussylvaticus*, *D.sylvestris* Ten. Fl. Neap. Prodr. (1811) p. xxv. - Eiusd. Fl. Nap. I (1811–1815) p. 231, Gargano, Tenore misit Nov 1827 / Herb. J. Gay., Presented by Dr. Hooker, February 1868, K000725365 (photo!).

#### Note.

In the same herbarium sheet three herbarium specimens, sent by Michele Tenore to Jaques Étienne Gay, are mounted. K000725363 was collected by Tenore from Calmaldoli (Campania, Italy) in November 1825; K000725364 by Nicolas Bové from La Calle (Algeria) in June 1839, and K000725365 by Tenore from Gargano (Apulia, Italy) in November 1827. In NAP there is a specimen from Gargano with the handwriting by Michele Tenore, lacking a date.

The lectotype designated here matches the protologue and corresponds to the current application of the name, which is considered as a heterotypic synonym of *D.tarentinus* Lacaita (Bacchetta et al. 2010; Brullo and Guarino 2017; Bartolucci et al. 2018). This synonymy is here confirmed based on the shape and size of the leaves, of the scales of the epicalyx and of the calyx which are observable on the types of the two taxa.

### ﻿ Dianthus virgineus var. tergestinus

Taxon classificationPlantaeCaryophyllalesCaryophyllaceae

Rchb., Icon. Fl. Germ. Helv. 6: 47, pl. 266 fig. 5049β?. 1842–1844. [1844 publ. 1842–1844]

E00D1489-A393-5BC1-99F9-A8F9892264FC

≡ D.tergestinus (Rchb.) A.Kern., Sched. Fl. Exs. Austro-Hung. 2: 71. 1883.
≡ D.caryophyllusvar.tergestinus (Rchb.) Tanfani in Caruel, Fl. Ital. 9(2): 283. 1892.
≡ D.sylvestrissubsp.tergestinus (Rchb.) Hayek, Repert. Spec. Nov. Regni Veg. Beih. 30(1, 2): 247. 1924. Ind. Loc.: none [but Trieste, Italy, can be easily inferred from the epithet “tergestinus” that means “from Trieste”].

#### Type.

(lectotype here designated): Rchb., Icon. Fl. Germ. Helv. 6: pl. 266 fig. 5049β. 1842–1844.

#### Note.

The main text (Icon. Fl. Germ. Helv. 6: 47. 1842–1844. [1844 publ. 1842–1844]) lacks a written diagnosis or description, and, in any case, it is not clear if the plate was published simultaneously with the main text. Stafleu and Cowan (1983) reports that the volume 6 was published between 1842 and 1844, even though the title page shows 1844. However, this name was validly published on plate CCLXVI (= 266) by an illustration with analysis (Arts. 38.7 and 38.8 of the ICN), which is obviously part of the original material.

This taxon is considered as a subspecies of *D.sylvestris* by Vangjeli (2015), Brullo and Guarino (2017), Bartolucci et al. (2018), Peruzzi et al. (2019), and Nikolić (2020). It differs from D.sylvestrissubsp.sylvestris by having a poorly developed mucro of the epicalyx scales and entire petals. Its distribution (Trieste area and along the north-eastern Adriatic coast), separated from the main range of D.sylvestrissubsp.sylvestris, is compatible with the rank of subspecies.

## Supplementary Material

XML Treatment for Dianthus aggericola

XML Treatment for Dianthus caryophyllus var. tenuifolius

XML Treatment for Dianthus caryophyllus f. minor

XML Treatment for Dianthus collivagus

XML Treatment for Dianthus consimilis

XML Treatment for Dianthus contractus var. evolutus

XML Treatment for Dianthus godronianus

XML Treatment for Dianthus orophilus

XML Treatment for Dianthus saxicola

XML Treatment for Dianthus juratensis

XML Treatment for Dianthus subacaulis

XML Treatment for Dianthus sylvestris

XML Treatment for Dianthus sylvestris var. garganicus

XML Treatment for Dianthus virgineus var. tergestinus
